# Medical management of cerebellar mutism syndrome at a quaternary children’s hospital

**DOI:** 10.1007/s00381-025-06759-8

**Published:** 2025-02-03

**Authors:** Emily Xu, Emily Zhang, Kristen Park, Mahaa Ayub, Chao Zhao, Jimmy W. Huh, J. Michael King, Iris Paltin, Amish C. Shah, Phillip B. Storm, Alexander Tucker, Peter J. Madsen, Shih-Shan Lang

**Affiliations:** 1 Division of Neurosurgery, Children’s Hospital of Philadelphia, Philadelphia, PA 19146 USA; 2 Department of Anesthesiology and Critical Care Medicine, Children’s Hospital of Philadelphia, University of Pennsylvania, Philadelphia, PA USA; 3 Division of Rehabilitation Medicine, Children’s Hospital of Philadelphia, Philadelphia, PA USA; 4 Division of Oncology, Children’s Hospital of Philadelphia, Philadelphia, PA USA

**Keywords:** Cerebellar mutism syndrome, Fluoxetine, Posterior fossa, Selective-serotonin reuptake inhibitors

## Abstract

**Purpose:**

We aimed to evaluate the efficacy of selective serotonin reuptake inhibitors (SSRIs) in treating cerebellar mutism syndrome (CMS).

**Methods:**

We retrospectively reviewed all pediatric patients who underwent a posterior fossa tumor resection between May 2007 to September 2022 at a single quaternary pediatric hospital. We evaluated clinical presentation and hospital course, including imaging findings, pathology, and surgical approaches. Propensity score matching was used to compare the symptom duration of patients who received SSRIs versus those who did not.

**Results:**

A total of 292 patients met the criteria with 25% (*n* = 73) being diagnosed with CMS. Several factors were significantly associated with a CMS diagnosis, such as pre-operative hydrocephalus (*p* = 0.002), a vermis-splitting approach (*p* = 0.007), tumor in the fourth ventricle (*p* = 0.010), medulloblastoma diagnosis (*p* = 0.009), and postoperative complication (*p* < 0.001). Of the patients diagnosed with CMS, 32.9% (*n* = 24) received SSRI treatment, specifically fluoxetine (*n* = 18) and sertraline (*n* = 6). Overall, treatment did not decrease the duration of CMS symptoms or shorten the inpatient rehab course compared to matched controls. However, within the cohort of fluoxetine-treated patients, earlier initiation of medication was significantly correlated with a shorter duration of mutism (*p* = 0.007).

**Conclusions:**

We report the largest cohort of CMS patients treated with SSRIs. The lack of overall clinical benefit when compared to untreated patients in our study may be due to the length of delay in starting an SSRI, since early initiation of fluoxetine correlated with shorter CMS symptoms. These results support the importance of early clinical detection of CMS and potentially treating CMS early in the patient’s postoperative course.

## Introduction

Cerebellar mutism syndrome (CMS), also known as posterior fossa syndrome or cerebellar cognitive affective syndrome, is a postoperative complication of posterior fossa tumor resection. It is most commonly seen in pediatric patients, occurring in around a quarter of cases [1], although adult cases have also been reported [2]. Symptoms normally develop within 1 to 2 days after surgery and include transient mutism, emotional lability, hypotonia, and ataxia [3]. Symptoms are typically transient and improve within a few months. However, several studies have shown persistent motor, neurological, and cognitive deficits for many years after surgery [4, 5].

The pathophysiology of postoperative CMS is still not understood but is hypothesized to result from damage to the efferent cerebellar pathway, specifically to the dentato-rubro-thalamic tract (DRTT). The neurons of the DRTT project from the dentate nuclei, traverse through the superior cerebellar peduncle, and synapse in the ventral lateral/ventral anterior nuclei of the thalamus with postsynaptic neurons that provide signals to the cortex. Data collected from MRI studies on patients with CMS have shown cerebellar edema that localizes to the superior cerebellar peduncle and the dentate nuclei [6]. Furthermore, functional MRI studies have demonstrated that CMS patients undergo connectivity disruptions in the periaqueductal grey that resolve once patients recover [7]. It is thought that through a mechanism termed diaschisis, DRTT injury causes loss of excitatory inputs to the motor, premotor, and prefrontal regions in the cortex, which would produce the movement and neurocognitive symptoms characteristic of CMS. Diaschisis explains how a primary injury can inhibit function in a remote area of the brain that is linked through neural pathways. Perfusion studies of CMS patients have suggested that hypoperfusion and hypometabolism of cortical regions are associated with cerebellar diaschisis [8], but the underlying parenchyma may also suffer permanent damage as well [9].

There are currently no standardized treatments for CMS, and management is often supportive. There have been a few case reports of pharmacological management, including the use of selective serotonin reuptake inhibitors (SSRIs). Early studies showed some promising potential of SSRIs, including fluoxetine and sertraline, in treating selective mutism, which is an anxiety disorder where a child fears speaking in settings where verbal communication is expected [10]. While the mutism in CMS has not been linked with anxiety, some groups have hypothesized that SSRIs could still be relevant for addressing the emotional lability component of the syndrome. However, only a few case reports have been published with no clear hypothesized mechanism of action [11–13].

In this study, we gather the largest cohort of treated CMS patients reported in the literature to date, and we evaluate whether SSRIs have a role in CMS management, comparing the symptom duration of CMS patients who received an SSRI with those who were untreated. We also add to the existing literature on preoperative risk factors for CMS development.

## Methods

### Study design

Data was retrospectively obtained from the electronic medical records of pediatric patients (< 18 years old) who underwent posterior fossa tumor resection between May 2007 and September 2022. All analyzed surgical cases were conducted by trained pediatric neurosurgeons at a single quaternary children’s hospital. Patients were selected to receive an SSRI if there was significant emotional lability and parental consent. Dosages were age- or weight-based (starting doses were between 2.5–10 mg fluoxetine and 12–25 mg sertraline in our cohort) and uptitrated as clinically indicated. SSRIs were recommended to be continued through the end of cancer treatment, but they were discontinued early if there was significant symptom improvement or resolution. The protocol was approved by the Committee for the Protection of Human Subjects Institutional Review Board (IRB).

### Variables

Patient records were retrospectively reviewed, and extracted data was inputted securely using Research Electronic Data Capture (REDCap) databases. Demographic data, hospital course, operative details, surgical complications, tumor pathology, and long-term outcomes, including duration of inpatient rehabilitation, physical therapy, occupational therapy, and speech therapy, were analyzed. Presence and severity of hydrocephalus were determined from pre-operative neuroimaging. Our primary outcome measure was the duration of primary CMS symptoms: mutism, gait impairment, fine motor impairment, dysphagia, and emotional lability. Mutism was defined as the complete absence of word production or sentences, but crying and/or whining could be produced [14]. Recovery of gait was measured by independent ambulation as defined by Function Ambulation Category ≥ 4 [15]. Fine motor ability was measured by independence in activities of daily living as scored by the Pediatric Evaluation of Disability Inventory, which includes evaluations on eating, grooming, bathing, and dressing [16]. Presence of dysphagia symptoms was evaluated by speech-language pathologists and by nasogastric or gastric tube use. Emotional lability was determined by clinical judgement. Symptoms were evaluated starting from postoperative day 1.

### Statistical analysis

Statistical analyses were performed using R statistical software (v4.3.0) and GraphPad Prism software. The study population was characterized by descriptive parameters of mean ± SD, median [Q1, Q3], and number (percentage). Odds ratios are reported with 95% confidence intervals. Continuous variables were compared using the Kruskal–Wallis test, and categorical variables were compared using the chi-squared test. Univariate analysis based on binary logistic regression was conducted to determine associated risk factors for CMS development. To minimize the effect of selection bias, propensity-score analysis was employed to match treated with untreated CMS patients. The following variables were used for 1:1 nearest neighbor matching: age, tumor size, tumor type, presence of a postoperative complication, and pre-operative hydrocephalus. A postoperative complication was defined as CNS infection, hemorrhage causing re-operation, postoperative hydrocephalus requiring re-operation, or redo resection within 1 week. The propensity score was estimated using logistic regression, and all standardized mean differences for the covariates were below 0.1, indicating adequate balance. All treated patients were successfully matched to yield 24 pairs of patients. The Kruskal–Wallis test was used to compare outcomes among the three treatment groups (none vs. fluoxetine vs. sertraline), and the Wilcoxon test was used for evaluating treatment pairs.

## Results

### Demographic factors

**Fig. 1:**
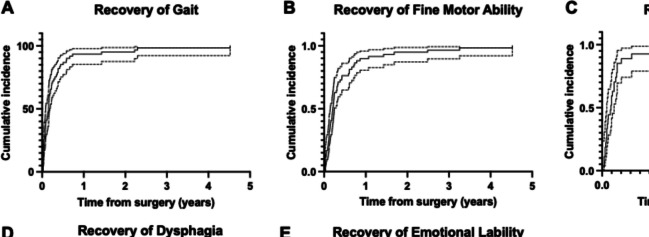
Cumulative incidence plots demonstrating the recovery of gait, fine motor ability, mutism, dysphagia, and emotional lability (**A–E**, respectively). Dotted lines indicate a 95% confidence interval

Demographics and surgical details are provided in Table 1. A total of 292 patients met the criteria with 25% (*n* = 73) being diagnosed with CMS. Among the patients with CMS, mutism was observed in 37% (*n* = 27) of patients, with a median duration of 17 [IQR: 9, 27] days (Fig. 1). Dysphagia was observed in 57.5% (*n* = 42) of patients, with 27 requiring a temporary nasogastric tube for an average of 29 weeks and 11 patients receiving a gastric tube. At the last follow-up, 2 patients still required a gastric tube. Median duration of dysphagia was 36 [IQR: 14,132] days. Emotional lability was present in 52% (*n* = 38) of patients. Most patients (*n* = 63) required inpatient rehabilitation after surgery for a median duration of 31 [IQR: 20, 62] days. Median follow-up time was 37 months.

**Table 1:** Patient demographics and surgical details

	Total (*n *= 292)	CMS patients (*n* = 73)
Average age in years at time of surgery ± standard deviation	7.86 ± 4.94	8.24 ± 4.5
Male	167 (57.2%)	38 (52.1%)
Tumor type		
Pilocytic astrocytoma	119 (40.8%)	32 (43.8%)
Medulloblastoma	81 (27.7%)	29 (39.7%)
Ependymoma	30 (10.3%)	7 (9.6%)
Atypical teratoid/rhabdoid tumor	12 (4.1%)	1 (1.4%)
Low grade glioma	10 (3.4%)	2 (2.7%)
Metastasis	7 (2.4%)	1 (1.4%)
Anaplastic astrocytoma	5 (1.7%)	0 (0)
High grade glioma	5 (1.7%)	0 (0)
Glioneuronal tumor	5 (1.7%)	0 (0)
Hemangioblastoma	4 (1.4%)	0 (0)
Diffuse intrinsic pontine glioma	3 (1%)	0 (0)
Schwannoma	2 (0.7%)	0 (0)
Pineoblastoma	2 (0.7%)	0 (0)
Choroid plexus papilloma	2 (0.7%)	0 (0)
Teratoma	1 (0.3%)	0 (0)
Germ cell tumor	1 (0.3%)	1 (1.4%)
Neuroblastoma	1 (0.3%)	0 (0)
Gliosarcoma	1 (0.3%)	0 (0)
Oligodendroglioma	1 (0.3%)	0 (0)
Tumor size (cm) ± standard deviation	4.3 ± 1.6	4.5 ± 1.5
Pre-operative hydrocephalus present	209 (71.6%)	63 (86.3%)
Severe hydrocephalus	65 (22.2%)	28 (38.4%)
Transependymal flow present	152 (52.1%)	52 (71.2%)
Surgical details		
Vermis-splitting approach	22 (7.5%)	11 (15.1%)
CUSA® use	221 (75.7%)	55 (75.3%)
Retractor use	228 (78.1%)	57 (78.1%)
Neuromonitoring use	96 (32.9%)	26 (35.6%)
Stereotactic navigation use	180 (61.6%)	51 (69.9%)
Postoperative CNS infection	9 (3.1%)	4 (5.5%)
Postoperative hemorrhage requiring re-operation	5 (1.7%)	2 (2.7%)
Postoperative hydrocephalus requiring re-operation	54 (18.5%)	21 (28.8%)
Re-do resection within 1 week	20 (6.8%)	11 (15.1%)
Postoperative cranial nerve injury	9 (3.1%)	3 (4.1%)
Average time to clinical detection of CMS symptoms (days) ± standard deviation		2.5 ± 3.9

### Associated risk factors

The odds of developing CMS were highest in the older 8–16 age range (OR = 6.75 [1.30, 166], *p* = 0.018) (Table 2). A diagnosis of medulloblastoma also conferred a higher risk of CMS (OR = 2.12 [1.21, 3.72], *p* = 0.009). CMS was most likely to develop in patients with a fourth ventricle tumor (OR = 2.02 [1.18, 3.45], *p* = 0.010) and least likely to develop from a tumor originating from the cerebellar hemisphere (OR = 0.34 [0.19, 0.61], *p* < 0.001). There were no differences in CMS development if there was an invasion into the brainstem (*p* = 0.363) or vermis (*p* = 0.312). However, a vermis-splitting surgical approach increased the risk of CMS (OR = 3.41 [1.41, 8.25], *p* = 0.007). Pre-operative hydrocephalus was also significantly associated with CMS development (OR = 3.15 [1.53, 6.50], *p* = 0.002), and those with severe hydrocephalus in particular were at even higher risk (OR = 3.57 [1.88, 6.81], *p* < 0.001).

**Table 2:** Associated risk factors for developing CMS

	OR [95% CI]	*p*-value
Surgery age (years)^‡^		
0–2	3.02 [0.46, 81.5]	0.282
2–8	4.86 [0.92, 121]	0.065
8–16	6.75 [1.30, 166]	**0.018**
Medulloblastoma	2.12 [1.21, 3.72]	**0.009**
Tumor location		
Cerebellar hemisphere	0.34 [0.19, 0.61]	** < 0.001**
Fourth ventricle	2.02 [1.18, 3.45]	**0.010**
Brainstem	0.67 [0.28, 1.59]	0.363
Vermis	1.33 [0.76, 2.32]	0.312
Pre-operative hydrocephalus	3.15 [1.53, 6.50]	**0.002**
Severe hydrocephalus	3.57 [1.88, 6.81]	** < 0.001**
Transependymal flow	2.15 [0.92, 4.98]	0.076
Postoperative complications*	3.02 [1.71, 5.32]	** < 0.001**
Vermis splitting approach	3.41 [1.41, 8.25]	**0.007**

^‡^Reference level > 16 years old

*Postoperative complications included central nervous system infection, hemorrhage causing re-operation, postoperative hydrocephalus requiring re-operation, or redo resection within 1 week

### Medical treatment

Out of the patients diagnosed with CMS, 32.9% (*n* = 24) received SSRI treatment, namely fluoxetine (*n* = 18) and sertraline (*n* = 6). The mean time between the onset of CMS symptoms and SSRI treatment initiation was 21 days (SD = 24 days). Using propensity score matching, the treatment group did not have a statistically significant difference in the duration of mutism (*p* = 0.972), dysphagia (*p* = 0.490), or emotional lability (*p* = 0.374) when compared to the untreated group. The patients given fluoxetine did have a longer impairment of gait (219.13 days vs. 114.83 days, *p* = 0.005) and fine motor abilities (250.75 days vs. 198.7 days, *p* = 0.032). The duration of in-patient rehabilitation (*p* = 0.679), occupational therapy (*p* = 0.647), speech therapy (*p* = 0.692), and physical therapy (*p* = 0.709) were not significantly affected by SSRI administration.

**Fig. 2:**
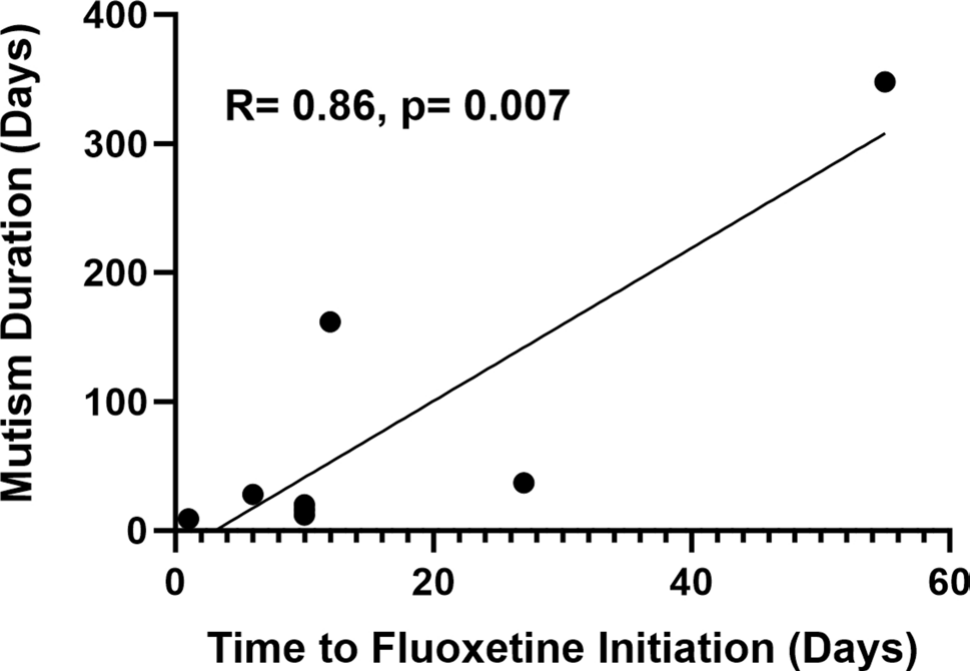
Relationship between timing of fluoxetine initiation and duration of mutism (*n* = 8). Line of best fit with correlation coefficient and *p*-value shown

Among the patients who received fluoxetine and also experienced mutism (*n* = 8), regression analysis between time to initiation of treatment and mutism length showed a statistically significant positive linear relationship (*R* = 0.86, *p* = 0.007) (Fig. 2). No statistically significant relationship was determined between fluoxetine initiation and gait impairment (*p* = 0.99), fine motor impairment (*p* = 0.18), dysphagia (*p* = 0.14), or emotional lability (*p* = 0.47) duration.

**Fig. 3:**
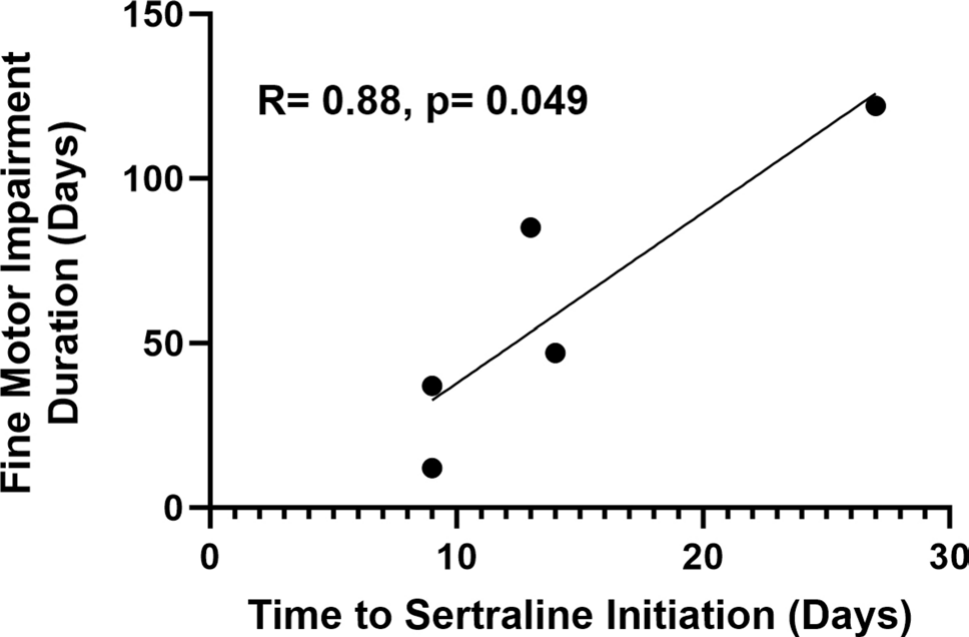
Time to initiation of sertraline correlated with fine motor impairment duration (*n* = 5). Line of best fit with correlation coefficient and *p*-value shown

For patients who received sertraline and experienced fine motor impairment (*n* = 5), a shorter time to starting treatment was correlated with a shorter duration of fine motor (*R* = 0.88, *p* = 0.0495) (Fig. 3) impairment. A similar relationship was seen with ambulation status, although it failed to reach statistical significance (*R* = 0.88, *p* = 0.051, *n* = 5). Regression analysis was precluded for mutism (*n* = 1) and dysphagia (*n* = 2) because not enough patients who received sertraline experienced those symptoms.

Side effects from fluoxetine were uncommon (*n* = 2). One patient experienced urinary retention but continued on the medication, while the other patient discontinued the medication because they developed a drug reaction with eosinophilia and systemic symptoms (DRESS) syndrome [17]. No side effects were reported for the patients who received sertraline.

## Discussion

This study aimed to evaluate the efficacy of SSRIs on CMS symptoms to identify whether treatment would ameliorate symptoms of this postoperative condition. We also report our single institutional experience with the potential risk factors for developing postoperative CMS.

In our cohort, 25% of the patients developed CMS, which is in line with previously reported studies [1, 18]. Additionally, our data confirms many of the established risk factors of CMS: midline location of the tumor, invasion of the fourth ventricle, incision of the vermis, and medulloblastoma diagnosis [19, 20]. Preoperative hydrocephalus has been a more controversial risk factor in the literature [19, 21]. In our study, it was found to be associated with CMS development. It is hypothesized that these risk factors predispose patients to midline damage of the efferent cerebellar pathways [22], and specifically to postsurgical swelling and disruption of the proximal DRTT, which has been implicated in motor coordination, cognition, and behavior [23]. Furthermore, MRI imaging of postoperative patients has suggested that bilateral, multi-focal damage to functional connections between the fastigial nuclei to the thalamus may be required for CMS development [7, 24, 25]. Thus, a midline insult has the highest likelihood of causing this pattern of injury. Medulloblastoma is a well-supported risk factor as it tends to develop in the midline and has a high incidence in the pediatric population. Work has been done to further explore risk factors of the molecular subgroups of medulloblastoma: Wingless (WNT), sonic hedgehog (SHH), Group 3, and Group 4. It was found that the SHH subgroup had the lowest risk of CMS, while Group 3 and Group 4 tumors were at the highest risk [20]. SHH tumors tend to arise from the cerebellar hemispheres, while Group 3 and Group 4 tumors tend to present in the fourth ventricle [20]. This finding further supports the theory that midline damage to the DRTT causes CMS and suggests a predictive method to identify medulloblastoma patients at risk of CMS based on molecular data.

The relationship between CMS and age is unclear. Some studies have found younger patients to be more susceptible to developing CMS [20], but others have found conflicting results [1]. In this study, patients between 8 and 16 years were at the highest risk. Additionally, while CMS is mainly thought of as a pediatric condition, there have been some adult cases reported in the literature. Comparisons between pediatric and adult CMS reveal a similar clinical course of transient mutism and midline cerebellar injuries as predisposing risk factors, supporting a common etiology of injury [26]. Therefore, all patients undergoing posterior fossa surgery should be monitored postoperatively for CMS symptoms, regardless of age.

Our study reports the largest group of CMS patients treated with a SSRI at a single institution. Overall, there was no shortening of CMS symptoms or duration of inpatient rehabilitation services with the treatment of a SSRI after propensity score matching. However, within the group of those who received treatment with fluoxetine, earlier initiation of fluoxetine was correlated with a shorter duration of mutism symptoms. Additionally, among the patients who received sertraline, there was a correlation between earlier sertraline initiation and faster motor recovery. Unfortunately, there were not enough patients to determine the effect of sertraline on mutism symptoms, so it is unclear whether the two drugs have different symptom targets. Sertraline is a more potent serotonin reuptake inhibitor than fluoxetine and also is able to bind to dopamine transporters, which may contribute to differing effects in CMS patients [27]. Our data suggest that SSRIs may be playing a role in recovery, but the potentially long lag time in initiation of treatment may have masked a difference when compared to the non-treatment group. There was an average window of 3 weeks from the onset of CMS symptoms to the start of SSRI treatment. In the case report by Akhaddar et al. [11], the 13-year-old patient received fluoxetine 3 weeks after CMS onset, and the patient’s mutism resolved after 4 weeks after starting fluoxetine. This is longer than the average 20.2 days of mutism in our untreated group.

The delay in starting a SSRI was often due to the difficulty of diagnosing CMS in the immediate postoperative period. There is frequent uncertainty between CMS and altered mental status from delayed general anesthesia effects, normal postoperative recovery for this location, ICU-related delirium, or postoperative pain. Furthermore, impairments are often more difficult to discern in the younger patients who have not fully progressed in their language and motor development. Finally, there may have been more caution in treating very young patients given the lack of evidence on SSRI side effects in this population.

To date, there have been no randomized clinical trials to evaluate pharmacologic therapies for CMS. Previous studies have suggested that SSRIs could be used for the symptoms seen in CMS. A few prospective trials [10, 28] have established their use in treating selective mutism. While the mutism in CMS is thought to be primarily due to a different mechanism than selective mutism, SSRIs could still provide symptomatic relief as it has been studied in many other conditions unrelated to mood or anxiety disorders. For example, SSRIs have been shown to promote motor and functional recovery in stroke patients [29]. Furthermore, fluoxetine has been studied in the treatment of emotional lability following vascular or traumatic brain injury, albeit with mixed results [30, 31]. There are several hypothesized mechanisms of action for how SSRIs can promote recovery after brain injury. While SSRIs exert their main anti-depressive effects by blocking serotonin reuptake in the synaptic cleft, they also exhibit additional neuroprotective effects after ischemic injury by suppressing the inflammatory response and increasing endothelial growth factor levels, which induce angiogenesis to support recovery [32, 33]. Serotonin has also been shown to induce new synaptic connections and increase the excitability of motor neurons [34]. Interestingly, in our study, neither fluoxetine nor sertraline showed significant efficacy in improving emotional lability symptoms. This may indicate that the emotional lability caused by CMS has a unique etiology from mood disorders.

The results of this study emphasize the need for a multidisciplinary team of neurosurgeons, critical care intensivists, neuro-oncologists, psychiatrists, and therapists to recognize and address physical, neurological, cognitive, and linguistic deficits. Patients with CMS are at increased risk of having residual speech, cognitive, and motor deficits at 1-year post-op [1]. Thus, many require extensive rehabilitation, physical and occupational therapy, speech therapy, and psychiatric and neuropsychological evaluations in order to have the best possible long-term outcomes. However, given the relatively safe pharmacological profile, our data supports early trialing of SSRIs once CMS symptoms are identified.

This study has some limitations. Given that SSRIs are not part of the standard of care for CMS, analysis was constrained by the small sample size of treated patients at a single center, limiting its generalizability to other institutions with different protocols. The purpose of this study is to serve as initial evidence to address a substantial gap in the literature and to spur further study with larger cohorts of patients. Additionally, because of the stigma around psychiatric medications in the pediatric population, treatment initiation and duration were influenced by parental preference. We attempted to mitigate these biases by using a matching protocol that controlled for several confounding variables and compared patients who had similar risk profiles, allowing us to isolate the effect of treatment even with a limited sample size. Finally, data for outpatient rehabilitation and duration of patient symptoms outside the immediate hospitalization was limited, so we were unable to assess the impact of SSRIs on long-term recovery.

## Conclusion

We report that earlier initiation of an SSRI was associated with a shorter duration of mutism symptoms with minimal side effects; therefore, patients undergoing posterior fossa surgery with predisposing risk factors should be monitored closely with the possibility of early trialing of an SSRI. Prospective trials are warranted to further assess the impact of different pharmacologic agents on CMS recovery.

## Data Availability

No datasets were generated or analysed during the current study.

## Abbreviations

CMS: Cerebellar mutism syndrome
DRESS: Drug reaction with eosinophilia and systemic symptoms
DRTT: Dentato-rubro-thalamic tract
REDCap: Research Electronic Data Capture Database
SNI: Severe neurologic impairment
SSRI: Selective serotonin reuptake inhibitor
